# Mother-Toddler Play Interaction in Extremely, Very Low Birth Weight, and Full-Term Children: A Longitudinal Study

**DOI:** 10.3389/fpsyg.2016.01511

**Published:** 2016-09-30

**Authors:** Paola Salvatori, Erica Neri, Ilaria Chirico, Federica Andrei, Francesca Agostini, Elena Trombini

**Affiliations:** Department of Psychology, University of BolognaBologna, Italy

**Keywords:** emotional availability, ELBW, VLBW, toddlers, play

## Abstract

**Introduction:** Although preterm birth represents a risk factor for early mother-infant interactions, few studies have focused on toddlerhood, an important time for the development of symbolic play, autonomous skills, and child's socialization competences. Moreover, no study has looked at the effect of birth weight on mother-child interactions during this period. Expanding on the available literature on prematurity, the main objective of this study was to explore the quality of mother-toddler interactions during play, using a longitudinal research design, as well as taking into account the effect of birth weight.

**Method:** 16 Extremely Low Birth Weight (ELBW), 24 Very Low Birth Weight (VLBW), 25 full-term children, and their mothers were recruited for the present study. Mother-child dyads were evaluated at 18, 24, and 30 months of child age. Ten minutes of mother-child play interaction were recorded and later coded according to the Emotional Availability Scales (EAS). Furthermore, the child's level of development was assessed through the Griffiths Scale, and its contribution controlled for.

**Results:** ELBW dyads showed an overall lower level of emotional availability, compared to VLBW and full-term dyads, but no main effect of birth weight was found on specific EA dimensions. Moreover, a significant effect of child age emerged. Overall scores, and Child Responsiveness and Involvement scores improved over time, independently of birth weight. Lastly, a significant effect of the interaction between birth weight and child age was found. Between 18 and 30 months, the overall quality of the interaction significantly increased in ELBW and VLBW dyads. Additionally, between 18 and 30 months, VLBW children significantly improved their responsiveness, while their mothers' sensitivity, structuring, and non-intrusive behaviors improved. In contrast, no change emerged in full-term dyads, although scores were consistently higher than those of the other groups.

**Discussion:** Birth weight affects the quality of mother-toddler interactions. Monitoring the relational patterns of preterm dyads during toddlerhood is important, especially in the case of ELBW children.

## Introduction

Infants are considered born prematurely when they are born alive before 37 weeks of pregnancy are completed (March of Dimes et al., 2012). The scientific community has defined different categories to identify the severity of prematurity, based on birth weight and on gestational age, with worse outcomes for infants weighing <1000 g and born before 32 weeks of gestation (Goldenberg et al., 2008; Saigal and Doyle, 2008). Prematurity represents a serious risk factor for several crucial domains of infant life, including development (Bhutta et al., 2002), maternal mental health (Voegtline et al., 2010; Agostini et al., 2014; Neri et al., 2015), and the quality of the mother-infant relationship (Bozzette, 2007; Korja et al., 2012; Bilgin and Wolke, 2015).

In this regard, a number of studies have found poorer mother-infant interactions in preterm dyads compared to full-term ones (Forcada-Guex et al., 2006; Bozzette, 2007; Feldman, 2007; Potharst et al., 2012). In particular, preterm infants are described as less responsive and less involved in the interaction with their mothers, showing less vocalizations, eye contact and more emotional negativity than full term infants (Davis et al., 2003; Singer et al., 2003; Montirosso et al., 2010; Korja et al., 2012). Mothers of preterm infants have, in turn, been described as more intrusive than those of full-term infants (Goldberg and DiVitto, 2002; Forcada-Guex et al., 2006, 2011; Bozzette, 2007; Feldman and Eidelman, 2007), while findings on maternal sensitivity provide a more mixed evidence. Specifically, although some studies found mothers of preterm infants to be less sensitive than mothers of full term infants (Forcada-Guex et al., 2006, 2011; Korja et al., 2012), others failed to find significant differences (Montirosso et al., 2010; Agostini et al., 2014; Rahkonen et al., 2014; Bilgin and Wolke, 2015).

In light of these findings, assessing other emotion-related variables, which may typify the dyadic interaction, such as the concept of emotional availability (Biringen and Easterbrooks, 2012), would prove helpful in better understanding the quality of preterm mother-child interactions. Emotional availability identifies the dyad's ability to share a healthy emotional connection, and describes the emotional range displayed by both the adult and the child during the interaction. It considers the dyadic relationship rather than focusing only on specific behaviors, which may be influenced by environmental or cultural biases, and fail to detect the overall quality of the relationship (Biringen and Easterbrooks, 2012; Saunders et al., 2015).

The severity of prematurity is a risk factor for the quality of dyadic exchanges between mother and child. In particular, the presence of birth complications (Singer et al., 2003; Muller-Nix et al., 2004), low gestational age (Sansavini et al., 2011), and extremely/very low birth weight (Neri et al., 2015) negatively affect the mother-child relationship. For example, preterm infants with a birth weight below 1500 g experience more difficult interactions with their mothers, including a higher level of maternal intrusiveness (Agostini et al., 2014; Neri et al., 2015), compared to infants with a birth weight above 1500 g. To our knowledge, however, very little research has explored the influence of birth weight in the context of dyadic interactions. Further studies on this topic could help increase available knowledge on preterm children's socio-emotional and cognitive developmental patterns (Righetti-Veltema et al., 2002; Beebe et al., 2011).

Furthermore, while most studies on prematurity have focused on the first year post-partum, little research has looked at mother-child interactions during toddlerhood (Forcada-Guex et al., 2006; Cho et al., 2008; Potharst et al., 2012; Salerni and Suriano, 2013).

A recent study by Salerni and Suriano (2013) found that preterm children, compared to full term children, showed lower levels of productivity during a play task with their mothers at 18 months, but not at 24 months. This study focused on symbolic play competences, but did not take into account other interactive patterns, such as the child's responsiveness and involvement, or maternal attitudes toward the child. To our knowledge, only one study, by Potharst et al. (2012), investigated maternal interactive behaviors during toddlerhood. This study showed that mothers of preterm children were less competent and supportive than those of full-term children, interfering with their child's autonomy, especially when the latter's development was impaired.

Despite the dearth of available studies, the second year of life represents an important period in child development, as it coincides with the development of new crucial competences, such as symbolic play, autonomous skills and active socialization (Piaget and Inhedler, 1969; McCune-Nicolich, 1981; Fonagy et al., 2001; Brazelton and Sparrow, 2006). Thus, it would be important to further explore whether the relational difficulties found during infancy also emerge at later ages.

The aim of the present study was, therefore, to explore emotional availability during play interactions between preterm children, in their second year of life, and their mothers. The rarely investigated effect of birth weight, and that of child age were taken into account. In line with literature, we hypothesized that lower birth weight would be associated with greater difficulties during mother-child play, despite a significant improvement in dyadic scores over time, both on the global level of emotional availability and on specific maternal and child behaviors.

Lastly, we explored the influence of the interaction between child age and birth weight on the quality of mother-child play interactions. Following our previous hypothesis, we expected an increase in the levels of emotional availability across all groups, but a greater improvement in full-term, compared to extremely, and very low birth weight dyads.

## Methods

### Participants

#### Sixty-five families participated in the study

Forty preterm infants with birth weight below 1500 g and their mothers were enrolled during the follow up program of the Neonatal Unit of the Bufalini Hospital in Cesena (Italy), between March 2013 and March 2014.

Inclusion criteria for preterm infants were: gestational age <37 weeks, birth weight ≤1500 g, and the absence of major cerebral damages (intraventricular hemorrhage III or IV grade, periventricular Leukomalacia, retinopathy of prematurity, and Hydrocephalus) and genetic syndromes. Inclusion criteria for parents were: being a normative parent, and having a Caucasian background.

All eligible families agreed to participate in the study. The Very Low Birth Weight (VLBW, birth weight between 1500 and 1000 g) group included 24 dyads, and the Extremely Low Birth Weight (ELBW, birth weight lower than 1000 g) group included 16 dyads.

Regarding the Control Group (CG), between April 2013 and April 2014, 60 mothers and their full term children were approached in preschools within the area of Cesena (Italy) and were asked to participate in the study. Inclusion criteria for full-term children were: gestational age >37 weeks, birth weight >higher than 2500 g, and the absence of cerebral damages and genetic syndromes. Inclusion criteria for CG parents were similar to those used for parents of preterm children.

Among the 60 full-term dyads initially approached, 34 mothers declined participation due to conflicting schedules. Of the 26 mothers who accepted to take part in the research, one dyad was excluded, due to major child health problems (i.e., epilepsy), hence, 25 dyads were included in the final CG.

### Procedure

The study was approved by the Ethical Committee of the Department of Psychology, University of Bologna (Italy). The recruitment of preterm dyads occurred at 15 months of child's corrected age during a follow-up visit at the Bufalini Hospital (Cesena, Italy), where a psychologist explained the objectives and the procedure of the study. The same psychologist also introduced the research to the mothers of the CG at their children's preschools, and scheduled the first appointment with those interested in participating in the study.

Data were collected at 18, 24, and 30 months of child age (corrected age for preterm children). All assessments were organized following the same procedure and lasted about 1 h each. During the first assessment, mothers gave their written informed consent, they completed a form regarding their socio-demographic information, and were asked data on their child's birth and history.

Ten to twenty minutes of dyadic interactions was then video-recorded, to assess the mother-child interaction. Since mother-child play is a valid method for assessing the dyadic relationship, mothers were asked to engage in a semi-structured play with their child using two dolls (one representing the mother, the other representing the child) and some play-dough. This methodology was inspired by the Doll-Play Technique, which is largely used in clinical studies, for its ability to access the child's and the mother's inner world through play narratives (Murray et al., 1999; Pass et al., 2012).

Lastly, since literature highlights how mother-child interactions might be more difficult in the context of impaired child development (Potharst et al., 2012; Rahkonen et al., 2014), the Griffiths Scales (Griffiths, 1996) were used to evaluate child competences, at each assessment, in order to control for its possible influence on mother-child interactions.

### Instruments

An *ad-hoc* questionnaire was created to collect infant information (gender, gestational age, length of hospitalization, twin status, type of delivery), and maternal socio-demographic data (e.g., age, nationality, marital status, education, occupation, parity). The socio-economic status (SES) of the family was calculated based on education and occupation of both parents, through Hollingshead's Index (Hollingshead, 1971).

All mother-child interaction videos were coded according to the Infancy/Early Childhood Version of the Emotional Availability Scales (4th edition) (EAS; Biringen, 2008). The EAS have been widely used in research, with both typical and atypical populations (Biringen, 2005; Wiefel et al., 2005; Saunders et al., 2015), including preterm infants (Zelkowitz et al., 2009; Patruno et al., 2015). They are used to measure the level of emotional availability in the parent-child relationship, which is defined as the emotional range displayed during the interaction by caregiver and child (Biringen and Easterbrooks, 2012). The EAS consist of six dimensions composed of seven subscales each. Four dimensions assess the adult's emotions and behavior (sensitivity, structuring, non-intrusiveness, non-hostility), and two focus on the child (responsiveness and involvement) (Table 1). A direct score is assigned for each dimension based on a 7-point Likert scale, with higher scores indicating more adequate interactive patterns.

**Table 1:** **EAS Dimensions (adapted by Biringen, 2008)**.

**Maternal scales**	
Sensitivity	Maternal ability to adequately respond to the child's cues during the interactions, and maternal positive affect. It includes the adult's positive affect, adequate perception of the child's emotions, acceptance of the child's behavior, flexibility, ability to handle conflicts, and awareness of timing.
Structuring	Maternal scaffolding capacity. It refers to the extent to which the adult is able to adequately guide the child during the interaction by taking care to follow the child's lead, setting limits for appropriate child behavior and/or misbehavior, establishing rules, and demanding compliance with rules. It takes into account both the provision of guidance, verbal and non-verbal, and the success of the adult's attempts.
Non-intrusiveness	Absence of over-directions, over-stimulations, interferences, or over protection in maternal behavior (i.e., commands, over-teaching, interferences with the child's play, verbal, or physical intrusion).
Non-hostility	Absence of covert or overt hostility towards the child. Hostile behavior includes verbal or physical aggressiveness like demeaning comments, impatience, boredom, critics, threats of separation, and introducing hostile play themes, or manipulating the child in a rough and violent way.
**Child scales**	
Responsiveness	Child's positive affectivity and appropriate responsiveness to the adult. It considers also the child's age-appropriate autonomy-seeking behavior, appropriate physical proximity to the adult, absence of role-reversal, lack of avoidance, and interest in the task of the play.
Involvement	Child's ability to actively engage with and involve the adult during the interactions. It evaluates the child's simple and elaborative initiative and takes into account the child's emotional or instrumental use of the adult, the lack of over-involvement, and the child's eye contact, body positioning, and verbal involvement with the adult.

In addition to the six dimensions, the EAS also offer an overall measure of caregiver-child emotional availability, through the EA Clinical Screener (Biringen, 2008). The EA Clinical Screener is an index of clinical relevance, which is assigned based on the overall interaction and describes the adult-child relationship on a 0–100 scale, giving a measure of relational risk: Scores between 100 and 81 indicate a healthy and emotionally available relationship (Emotionally Available); scores between 70 and 61 refer to a complicated relationship characterized by inconsistent adult and child behavior, such as pseudo-sensitivity in the adult, and negative attention seeking behaviors, dependency or over-connection in the child (Complicated Emotional Availability); scores between 60 and 41 indicate an avoidant relationship (Emotionally Unavailable/Detached); scores between 40 and 1 identify a very problematic, possibly traumatized relationship (Problematic/Disturbed) (Biringen, 2008). In line with literature (Licata et al., 2014, 2016; Baker et al., 2015) we used the Clinical Screener scores as a continuous variable, to assess the global level of mother-child emotional availability during play.

Two blind raters (P.S. and M.M.), trained to reliability in the use of the EAS, through the Biringen on-line EA training, coded all the videos. Both raters completed the criterion/reliability cases for the 4th edition of the Emotional Availability Scales, and achieved an acceptable level of reliability with the author. The degree of agreement between the two coders was measured on a random selection of 30% of the videos. The intraclass correlation coefficient between the two coders was found to be good for research purposes and ranged between 0.70 and 0.86 (mean = 0.80).

Child development was assessed using the Griffiths Mental Development Scales (GMDS, Griffiths, 1996), a well-recognized tool for measuring infant mental and psychomotor development in the clinical follow-up of preterm infants (Giannì et al., 2007; Biasini et al., 2012; Agostini et al., 2014; Neri, 2016). The scales evaluate six specific areas of child development: locomotor, personal-social, hearing and language, eye-hand co-ordination, performance and practical reasoning. Higher scores on each scale correspond to a better development in a specific cognitive domain. For each scale, percentile scores can be computed from raw scores. Percentile scores are sensitive measures to assess child improvement over time in chronic disorders, or neonatal follow-up programs (Griffiths, 1996).

### Statistical analysis

Statistical analysis was performed using IBM SPSS version 20.0 for Windows. A *p* < 0.05 was considered significant.

Descriptive analyses were run in order to verify homogeneity of the sample on socio-demographics and obstetrical variables (Pearson's *X*^2^ Test, and Univariate ANOVA).

The effects of Birth Weight (ELBW, VLBW, and CG), Child Age (18, 24, and 30 months), and their interaction, on the EA Clinical Screener continuous scores and on the EA dimensions were then tested through Linear Mixed Models (LMMs, two levels, with random intercept). Bonferroni's *post-hoc* test was used for pairwise comparisons.

## Results

### Descriptive statistics

The maternal and child socio-demographic characteristics are shown in Table 2.

**Table 2:** **Infant and maternal socio-demographic and clinical characteristics**.

	**ELBW group (*N* = 16)**	**VLBW group (*N* = 24)**	**Control group (*N* = 25)**	***F/X*^2^**	***p***
**INFANT CHARACTERISTICS**					
**Gender**					
Male, n (%)	5 (31.2)	12 (50.0)	15 (60.0)	3.235	0.198
Female, n (%)	11 (68.8)	12 (50.0)	10 (40.0)		
Gestational Age—weeks, m (sd)	27.67 (1.42)	30.13 (2.48)	39.67 (1.23)	245.653	<0.0001
Length of hospitalization- days, m (sd)	58.00 (13.74)	35.35 (11.50)	//	30.211	<0.0001
**Twin status**					
Yes, n (%)	1 (6.2)	11 (45.8)	0 (0.0)	19.190	<0.0001
No, n (%)	15 (93.8)	13 (54.2)	25 (100.0)		
**Type of delivery**					
Spontaneous, n (%)	4 (25.0)	3 (12.5)	18 (72.0)	19.940	<0.0001
Cesarean, n (%)	12 (75.0)	21 (87.5)	7 (28.0)		
GMDS general percentile score, m (sd)	54.12 (4.44)	55.34 (3.48)	59.83 (3.38)	0.670	0.515
**MATERNAL CHARACTERISTICS**					
Age, years, m (sd)	36.36 (7.62)	37.71 (3.90)	36.50 (4.86)	0.422	0.658
Hollingshead SES score, m (sd)	32.14 (18.23)	43.02 (15.87)	42.60 (16.14)	1.870	0.164
**Nationality**					
Italian, n (%)	10 (62.5)	24 (100.0)	24 (96.0)	15.986	<0.0001
Foreign, n (%)	6 (37.5)	0 (0.0)	1 (4.0)		
**Marital status**					
Married/Cohabiting, n (%)	12 (75.0)	24 (100.0)	25 (100.0)	13.053	0.001
Other, n (%)	4 (25.0)	0 (0.0)	0 (0.0)		
**Education**					
University, n (%)	3 (15.4)	13 (52.2)	19 (75.0)	16.979	0.002
High school, n (%)	7 (46.2)	10 (43.5)	4 (16.7)		
Primary/Secondary school, n (%)	6 (38.5)	1 (4.3)	2 (8.3)		
**Occupation**					
Employed, n (%)	13 (80.0)	24 (100.0)	23 (91.7)	5.062	0.080
Unemployed, n (%)	3 (20.0)	0 (0.0)	2 (8.3)		
**Parity**					
Nulliparous, n (%)	11 (68.8)	19 (79.2)	21 (84.0)	1.354	0.508
Multiparous, n (%)	5 (31.2)	5 (20.8)	4 (16.0)		

The three groups were homogenous with respect to child gender, maternal age, parity, occupation, family socio-economical level, and child global development level (Table 2).

Significant differences among groups emerged in gestational age [*F*_(2, 62)_ = 245.657, *p* < 0.0001], length of hospitalization [*F*_(1, 36)_ = 30.211, *p* < 0.0001], and twin birth [*X*^2^_(1)_ = 19.190, *p* < 0.0001] (Table 2). These results were expected since these variables are strictly linked to premature birth.

With regards to maternal variables, differences were found in nationality [*X*^2^_(2)_ = 15.986, *p* < 0.0001], education [*X*^2^_(4)_ = 16.979, *p* = 0.002], and marital status [*X*^2^_(2)_ = 13.053, *p* = 0.001] (Table 2). Subsequent analyses showed that nationality and marital status did not significantly influence EAS scores. For this reason, these variables were not included in further analyses. On the contrary, education showed a significant association with the EAS and was, therefore, controlled in subsequent analyses.

In addition, taking into account previous literature showing the importance of child gender for early socio-emotional development, we investigated its effect on EA scales. No significant effect emerged on the Clinical Screener scores [*F*_(1, 192)_ = 0.355, *p* = 0.55], or on any of the EA dimensions: Sensitivity [*F*_(1, 192)_ = 0.442, *p* = 0.51]; Structuring [*F*_(1, 192)_ = 1.846, *p* = 0.18]; Non-Intrusiveness [*F*_(1, 192)_ = 0.036, *p* = 0.85]; Non-Hostility [*F*_(1, 192)_ = 0.0001, *p* = 0.99]; Child Responsiveness [*F*_(1, 192)_ = 0.150, *p* = 0.70]; Child Involvement [*F*_(1, 191)_ = 0.012, *p* = 0.91]. As no significant difference emerged, we did not further control for this variable.

### Mother-child interactions (EAS)

LMMs were run for the EA Clinical Screener, and for each EA dimension, in order to test the main effects of birth weight, child age and their interaction on mother-child play. The level of maternal education was always included as a control variable.

#### Birth weight

A main effect of Birth Weight on the EA Clinical Screener scores emerged [*F*_(2, 53.703)_ = 9.805, *p* < 0.0001] (Table 3). *Post-hoc* tests showed significantly higher scores in CG dyads, compared to VLBW (*p* = 0.028), and ELBW dyads (*p* < 0.0001).

**Table 3:** **Mother-child interactive behaviors (EAS): differences among groups**.

	**Birth weight**			**Child age**			***F***		
	**ELBW (*N* = 16)**	**VLBW (*N* = 24)**	**CG (*N* = 25)**	**18 Months**	**24 Months**	**30 Months**	**Birth weight**	**Child age**	**Birth weight × Child age**
EA clinical screener	70.391 (2.8)	79.181 (2.3)	87.025 (2.4)	75.757 (1.6)	78.930 (1.6)	81.910 (1.6)	9.805^**^	17.624^**^	3.122^*^
**EA DIMENSIONS**									
**Maternal scales**									
Sensitivity	5.195 (0.29)	5.199 (0.23)	5.503 (0.22)	5.253 (0.17)	5.228 (0.17)	5.415 (0.17)	0.675	1.581	2.667^*^
Structuring	5.190 (0.30)	5.040 (0.24)	5.241 (0.24)	5.071 (0.19)	5.027 (0.19)	5.372 (0.19)	0.223	2.772	2.658^*^
Non-intrusiveness	5.149 (0.27)	5.195 (0.21)	5.538 (0.21)	5.241 (0.17)	5.228 (0.17)	5.414 (0.17)	1,068	1.022	4.683^**^
Non-hostility	6.320 (0.18)	6.298 (0.14)	6.427 (0.14)	6.223 (0.11)	6.373 (0.12)	6.449 (0.12)	0.279	2.632	0.812
**Child scales**									
Responsiveness	4.918 (0.26)	5.204 (0.20)	5.488 (0.20)	5.007 (0.16)	5.129 (0.16)	5.474 (0.17)	1.749	6.261^**^	3.267^*^
Involvement	5.451 (0.26)	4.974 (0.21)	5.053 (0.21)	4.346 (0.16)	4.878 (0.17)	5.253 (0.17)	1.953	21.379^**^	0.916

* *p < 0.05*;

** *p < 0.005*.

No main effect of birth weight on specific EA dimensions was found (Table 3).

#### Child age

A main effect of Child Age on the EA Clinical Screener scores also emerged [*F*_(2, 102.782)_ = 17.624, *p* < 0.0001] (Table 3): scores significantly increased from 18 months to 24 (*p* = 0.007) and 30 months (*p* < 0.0001), and from 24 to 30 months (*p* = 0.015).

With regards to the specific EA dimensions, main effects of Child Age emerged on Child Responsiveness [*F*_(2, 103.254)_ = 6.261, *p* = 0.003] and Involvement [*F*_(2, 104.526)_ = 21.379, *p* < 0.0001] (Table 3). For Child Responsiveness, 30 months scores were higher than 18 (*p* = 0.002), and 24 month ones (*p* = 0.041). Lower scores on the Child Involvement scale were found at 18 months compared to 24 (*p* < 0.0001) and 30 months of age (*p* < 0.0001), and at 24 months compared to 30 months (*p* = 0.029). We found no effect of Child Age on maternal Sensitivity, Structuring, Non-Intrusiveness and Non-Hostility scales.

#### Birth weight and child age

A significant effect of the interaction between Birth Weight and Child Age was found on the EA Clinical Screener scores [*F*_(4, 102.767)_ = 3.122, *p* = 0.018] (Table 3). Specifically, at 18 months, scores of ELBW dyads were lower than those of VLBW (*p* = 0.029) and CG (*p* < 0.0001) dyads, and VLBW dyads showed lower scores than CG dyads (*p* = 0.022). At 24 months, ELBW dyads showed lower scores than VLBW (*p* = 0.017) and CG (*p* < 0.0001) dyads. At 30 months, however, ELBW scores were only lower than CG ones (*p* = 0.019).

**Figure 1:**
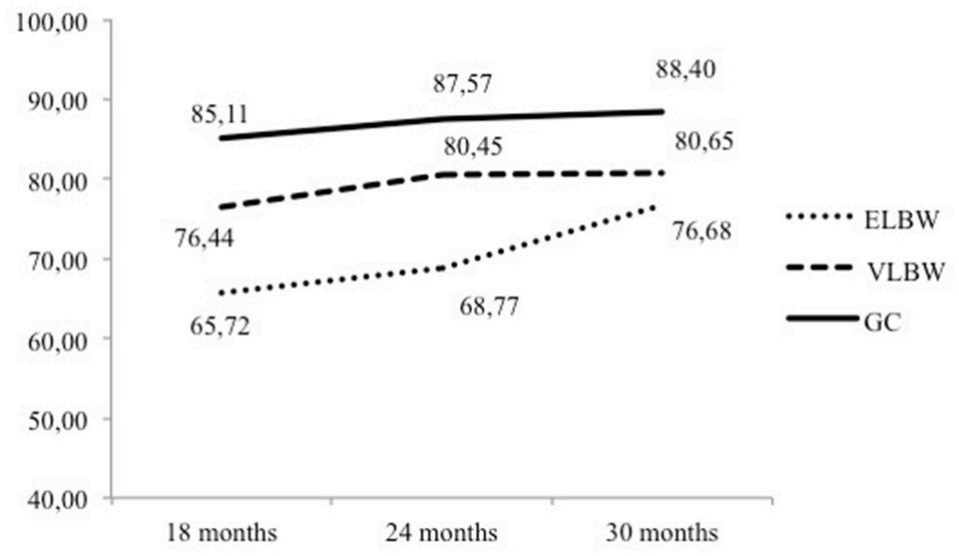
**Interaction between birth weight and child age on the EA clinical screener scores**.

When differences within groups were considered, in the ELBW group, scores were significantly higher at 30 months compared to 18 months (*p* < 0.0001) and 24 months (*p* = 0.001), whereas VLBW scores increased from 18 months to 24 (*p* = 0.034) and 30 months (*p* = 0.041) (Figure 1). No change over time was instead found in the full-term group, where scores showed substantial stability across all time points (*p* > 0.05).

**Figure 2:**
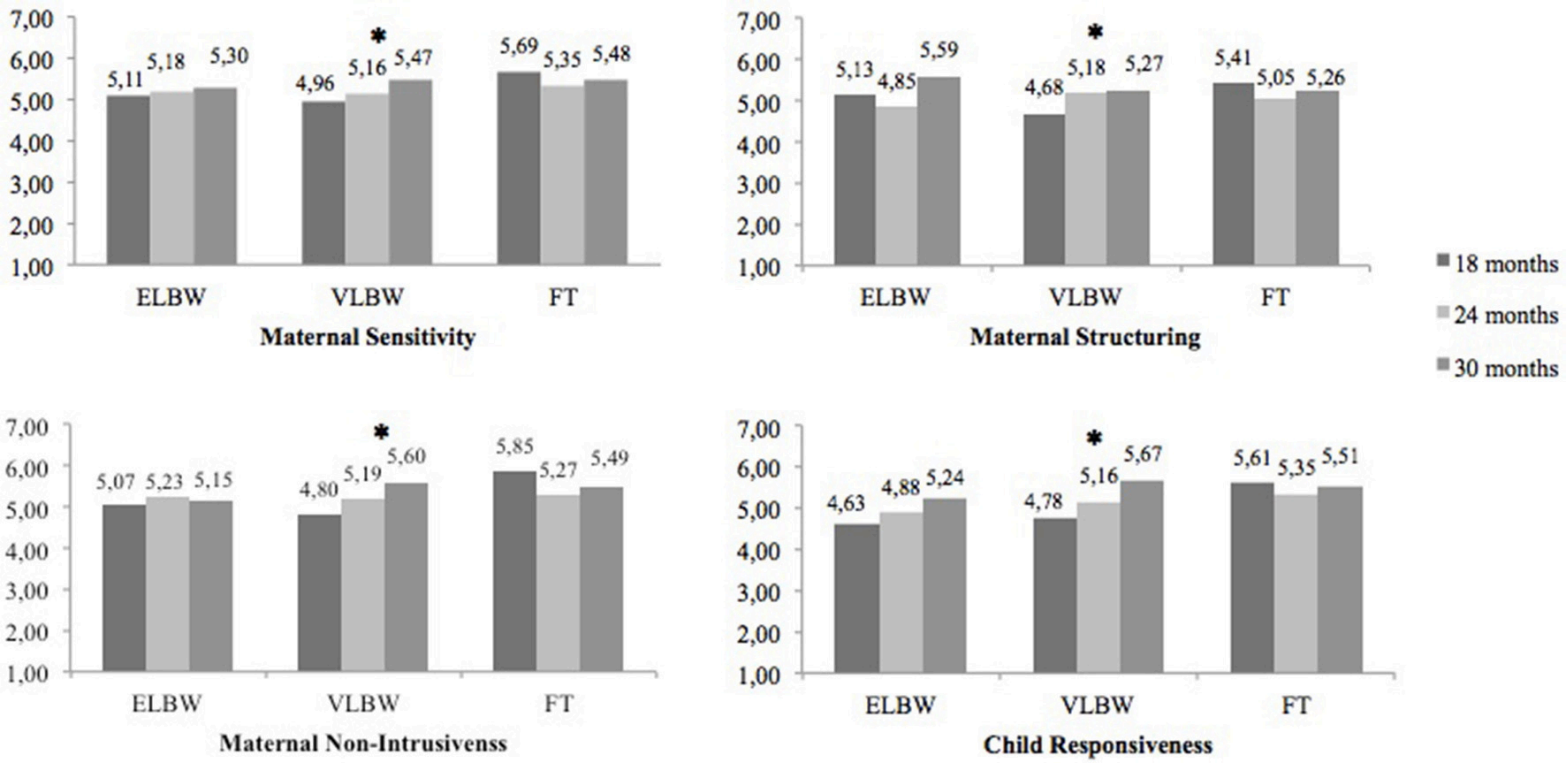
**Interaction between birth weight and child age on maternal sensitivity, maternal structuring, maternal non-intrusiveness, and child responsiveness scale**. *Stands for significant effects with *p* < 0.05.

The interaction between Birth Weight and Child Age was also significant for the following EA dimensions: Sensitivity [*F*_(4, 103.938)_ = 2.667, *p* = 0.036], Structuring [*F*_(4, 104.996)_ = 2.658; *p* = 0.037], Non-Intrusiveness [*F*_(4, 107.037)_ = 4.683, *p* = 0.002], and Child Responsiveness [*F*_(4, 102.982)_ = 3.267, *p* = 0.014] (Table 3). Only in the VLBW group, the scores significantly increased from 18 to 30 months on maternal Sensitivity (*p* = 0.013), Structuring (*p* = 0.050), Non-Intrusiveness (*p* = 0.002) and Child Responsiveness (*p* < 0.0001), whereas no significant change was found in full-term or ELBW dyads (Figure 2).

No significant difference emerged in Non-Hostility and Child Involvement (Table 3).

#### Education

No effect of maternal education emerged on the EA clinical screener scores [*F*_(2, 53.610)_ = 1.328, *p* = 0.274].

An effect of maternal education was, however, found on Sensitivity [*F*_(2)_ = 3.785, *p* = 0.029], Non-Intrusiveness [*F*_(2)_ = 4.740, *p* = 0.013], Non-Hostility [*F*_(2)_ = 4.111, *p* = 0.022], and Child Responsiveness [*F*_(2)_ = 3.741, *p* = 0.030]: highly educated mothers (university degrees), compared to those with secondary school education, appeared more sensitive (*p* = 0.024), less intrusive (*p* = 0.01), and hostile (*p* = 0.018) and their infants were more responsive (*p* = 0.032). The latter mothers also showed lower Child Responsiveness scores than mothers with high school education (*p* = 0.048).

## Discussion

The main aim of the present study was to explore mother-child emotional availability during play in the second year of life comparing ELBW, VLBW, and full-term dyads.

Our first hypothesis, concerning the effect of birth weight on mother-child emotional availability, was only partially confirmed. On one hand, results showed that the ELBW group had the lowest scores on the Clinical Screener index and the full-term group the highest. The quality of the interaction, therefore, seems to worsen proportionally with the severity of birth weight, confirming our hypothesis. On the other hand, however, no difference on individual EA dimensions emerged among groups, in contrast to what we expected. This result raises questions on the methods used to assess mother-child interactions in the context of prematurity. The present findings seem to suggest that, during the second year of life, differences in mother-child interactions among birth weight groups may be more evident when interactions are considered globally rather than when focusing on specific maternal and child interactive behaviors. This explanation would be coherent with other studies, including a recent meta-analysis (Bilgin and Wolke, 2015), which failed to find differences between preterm and full-term groups in specific maternal patterns. Furthermore, it is possible that the interactive patterns of preterm dyads could improve over time, and that the differences observed at earlier ages (Forcada-Guex et al., 2006; Bozzette, 2007; Feldman, 2007; Korja et al., 2012) become less evident during toddlerhood.

Such explanation is in line with our findings, which showed that, across time, all groups had an increase in child responsiveness and involvement and in the overall quality of the interaction, thus supporting our second hypothesis. This could be explained by the fact that toddlerhood is a period characterized by many acquisitions (symbolic play, autonomous skills, and active socialization), which are shown by the child through the progressive development of a skilled play behavior (Piaget, 1972; Brazelton and Sparrow, 2006). Our results might reflect the natural evolution of the children's relational competences, which allow them to be more engaged during play. Considering the importance that age seems to play on child interactive behaviors, broader longitudinal studies should be carried out, to explore the evolution of child relational patterns across developmental stages, from infancy to toddlerhood.

Our second hypothesis was instead not confirmed in relation to EAS maternal dimensions. Maternal attitudes toward the child might characterize by a greater stability, as already highlighted by other studies (Bornstein et al., 2006; Stack et al., 2012).

Our findings related to the interaction between Child Age and Birth Weight, related to our third hypothesis, showed the presence of group differences in the development of mother-child interactions over time. Full-term group scores did not improve over time, in contrast to what we had hypothesized, although they remained higher than those of VLBW and ELBW dyads. Notably, a significant improvement in the overall level of mother-child emotional availability was instead seen in the VLBW and ELBW groups. Furthermore, we found an increase in maternal sensitivity, structuring, non-intrusiveness, and child's responsiveness in the VLBW group from 18 to 30 months. These results seem to indicate that, although, initially, VLBW and ELBW dyads experience more difficulties in mother-child interactions, in line with literature findings (Korja et al., 2012; Agostini et al., 2014; Neri et al., 2015), they seem to reach more adequate levels of emotional availability at the end of the second year of life. The second year of life might, therefore, represent a pivotal time for preterm dyads in their adjustment to the consequences of prematurity. Full-term dyads, instead, might have already found an adjustment, thus showing more stability in their behaviors throughout the second year of life. This explanation seems to be consistent with a recent Italian study by Salerni and Suriano (2013). This study showed that preterm children increased their playing abilities from 18 to 24 months of corrected age, whereas full-term children did not show a significant improvement. The authors hypothesized that this phenomenon could be related to full-term children being advantaged by having already reached adequate interactive and play skills at 18 months. Preterm children, on the other hand, showing a delayed start compared to their full-term counterparts, had to catch up. Our study adds to these findings, by showing how the effect of prematurity might vary according to the child's birth weight, with only VLBW dyads showing an improvement in maternal sensitivity, structuring, non-intrusiveness, and child responsiveness. Furthermore, while VLBW dyads showed an improvement in overall mother-child emotional availability as early as between 18 and 24 months, ELBW dyads only showed this between 24 and 30 months. This might suggest that ELBW dyads need more time to adjust to the traumatic effect of premature birth, compared to VLBW. These findings seem to support the idea that ELBW dyads may struggle more than VLBW dyads, due to the greater fragility of these children. With regards to this, previous studies (Stern and Karraker, 1990; Stern et al., 2006; Patruno et al., 2015) have shown how mothers of preterm children tend to have a stereotyped perception of their infants, seeing them as weak and vulnerable. This perception, which can affect the quality of mother-child interactions, could be stronger, and more persistent in time, in mothers of ELBW children, compared to VLBW ones. Further investigations should be conducted to confirm this explanation, and future studies should consider maternal perception of the child when evaluating mother-child interactions in the context of prematurity.

With respect to the individual EA dimensions, it is interesting to note that Non-Hostility scores, in contrast to the other dimensions, were high for all groups and at all time points, and variability was lower than in other scales. This could be due to several reasons: A first possible explanation is that a low-stress context of observation like the one used in our study may have reduced the possibility of detecting hostile maternal behaviors. This would be consistent with a recent review by Biringen et al. (2014), which highlighted the small number of studies reporting significant findings on Non-Hostility, due to the limited use of stressful contexts of observation, or of periods of observation long enough to capture moments of stress or loss of control in the adult. In relation to this, a second reason might be linked to the duration of mother-child interactions. In line with literature and the EAS guidelines (Biringen, 2008), mother-child interactions in our study were videotaped for 10–20 min, yet it is possible that this length of time was not enough to observe a stressful situation in a low-risk sample, such as the one examined. As shown in other studies, high levels of maternal hostility are often associated with high-risk contexts, such as disadvantaged communities, previous histories of abuse and maltreatment, or of substance abuse (Little and Carter, 2005; Bornstein et al., 2006; Moehler et al., 2007; Stack et al., 2012; Porreca et al., 2016). Our sample was mostly composed of middle-class women, living or cohabiting with their child's father, and without any diagnosed psychiatric disorder or major health issue. Hence, both the study setting and the characteristics of our sample might have limited the possibility of detecting significant differences and associations related to Non-Hostility scores. Compared to situations of higher risk, with low risk samples, a longer duration of the observation or the use of more specific instruments could help to better detect differences in the levels of hostility displayed by mothers.

Overall, these results seem to underline how VLBW and ELBW dyads reached more adequate levels of emotional availability at the end of the second year of life. However, it is noteworthy that the specific characteristics of the sample, which only included healthy preterm children (without cerebral damages or syndromes), may have biased these results. Moreover, all preterm dyads were recruited during the follow-up program of the Bufalini Hospital (Cesena, Italy), a non-structured intervention during which parent-child interactions were assessed and psycho-educational recommendations were given to promote adequate parental behaviors. Results on the Griffiths scales seem to support the positive effects of the program, given the absence of developmental difference among birth weight groups. Hence, this intervention might have positively influenced the quality of the dyadic play observed in this study. Future research should explore the direct effect of follow-up programs on mother-child play interactions.

Finally, our study detected an effect of maternal education on dyadic interactive behaviors. Although investigating the effect of education was not an objective of this study, this effect is consistent with literature findings (Holditch-Davis et al., 2007; Bigelow et al., 2010; Potharst et al., 2012), and underlines the need of considering maternal characteristics in future studies.

The present study has a number of strengths, including its focus on the effect of birth weight and on toddlerhood, a period so far poorly investigated, and its use of the Doll-Play methodology, with its rich evaluation of the relationship between the mother and the child. The structured context of play, in fact, elicits the expression of maternal and child internal representations during the interaction (Murray et al., 1999; Pass et al., 2012).

Although our study adds relevant and promising results to the literature, some limitations should be considered. First, due to the small sample size, further investigations on wider samples should be undertaken to confirm our findings. Moreover, while this study only focused on the role of mothers during play, recent literature on early interactions shows the relevance of fathers for child development. Therefore, future studies should extend our results by taking into consideration father-child interactions, during the second year of life. Finally, as maternal depression and anxiety could influence maternal interactive behaviors during the first year after preterm birth (Zelkowitz et al., 2009; Vigod et al., 2010; Neri et al., 2015), such variables should be taken into account and controlled for by future studies.

The findings here reported have important clinical implications, as they suggest how special attention should be paid to mother-child interactions in ELBW and VLBW dyads during the second year of life, a period that represent a pivotal time for the adjustment to preterm birth. Monitoring the quality of dyadic interactions is important to support and enhance the onset and structuring of the relationship between mother and child.

## Author contributions

PS: Conceptualized and designed the study, included participants, acquired, analyzed and interpreted data, drafted and reviewed the initial and final manuscript as submitted. She declared that she have not competing interest. EN: Acquisition, analyses and interpretation of data, reviewed and approved the final manuscript as submitted. She declared that she have not competing interest. FeA: Took part in the design, analyzed and interpreted data, gave technical support and advice, reviewed and approved the final manuscript as submitted. She declared that she have not competing interest. IC: Took part in the design, analyzed and interpreted data, gave technical support and advice, reviewed and approved the final manuscript as submitted. She declared that she have not competing interest. FrA: Acquisition, analyses and interpretation of data, reviewed and approved the final manuscript as submitted. She declared that she have not competing interest. ET: Conceptualized and designed the study, included participants, acquisition, analyzed and interpreted data, reviewed and approval the final manuscript as submitted. She declared that she have not competing interest.

### Conflict of interest statement

The authors declare that the research was conducted in the absence of any commercial or financial relationships that could be construed as a potential conflict of interest.
